# Mechanisms of Genome Instability in the Fragile X-Related Disorders

**DOI:** 10.3390/genes12101633

**Published:** 2021-10-17

**Authors:** Bruce E. Hayward, Karen Usdin

**Affiliations:** Section on Gene Structure and Disease, Laboratory of Cell and Molecular Biology, National Institute of Diabetes, Digestive and Kidney Diseases, National Institutes of Health, Bethesda, MD 20892, USA; bruce.hayward@nih.gov

**Keywords:** repeat mosaicism, chromosome fragility, repeat expansion, repeat contractions, aneuploidy, break induced replication (BIR), base excision repair (BER), microhomology mediated end-joining (MMEJ), mitotic DNA synthesis (MiDAS)

## Abstract

The Fragile X-related disorders (FXDs), which include the intellectual disability fragile X syndrome (FXS), are disorders caused by expansion of a CGG-repeat tract in the 5′ UTR of the X-linked *FMR1* gene. These disorders are named for FRAXA, the folate-sensitive fragile site that localizes with the CGG-repeat in individuals with FXS. Two pathological *FMR1* allele size classes are distinguished. Premutation (PM) alleles have 54–200 repeats and confer the risk of fragile X-associated tremor/ataxia syndrome (FXTAS) and fragile X-associated primary ovarian insufficiency (FXPOI). PM alleles are prone to both somatic and germline expansion, with female PM carriers being at risk of having a child with >200+ repeats. Inheritance of such full mutation (FM) alleles causes FXS. Contractions of PM and FM alleles can also occur. As a result, many carriers are mosaic for different sized alleles, with the clinical presentation depending on the proportions of these alleles in affected tissues. Furthermore, it has become apparent that the chromosomal fragility of FXS individuals reflects an underlying problem that can lead to chromosomal numerical and structural abnormalities. Thus, large numbers of CGG-repeats in the *FMR1* gene predisposes individuals to multiple forms of genome instability. This review will discuss our current understanding of these processes.

## 1. Introduction

In the late 1940s, J. Purdon Martin and Julia Bell described an extended family with 13 cases of intellectual disability and an unusual pattern of X-linked inheritance in which the traits could be transmitted by males who displayed no clinical symptoms [1] to males in later generations who did. It was more than 20 years later that a “marker X chromosome”, showing the chromosome abnormality we now call a fragile site, was first shown to co-segregate with these traits [2]. Originally known as Martin–Bell syndrome, the fragile site eventually gave this disorder the name we know it by today, fragile X syndrome (FXS: OMIM # 300624). The fragile site, FRAXA, a chromosome constriction, gap, or break is seen at the end of the long arm of the X chromosome in metaphase cells subjected to folate-stress. The site became an important diagnostic marker for FXS that was used until the early 1990s, when the gene responsible for this disorder was identified and Southern blotting became the gold standard for diagnosis. The most common mutation associated with FXS is a large and unstable CGG-repeat tract in the 5′ UTR of the *FMR1* gene (MIM* 309550) [3,4]. *FMR1* encodes FMRP, an RNA-binding protein important for learning and memory. The repeat tract, which is coincident with the fragile site at Xq27.3, explains the unusual inheritance pattern that had become known as the Sherman paradox; the repeat is expansion prone, tending to gain repeats with each successive generation, with symptoms of FXS only becoming apparent when the repeat number exceeds 200 [5]. Such alleles are known as full mutation (FM) alleles and the symptoms of this disorder arise because the FM allele undergoes a process of repeat-mediated gene silencing that results in a deficit of FMRP [6]. FM alleles originate from maternally transmitted alleles with 54–200 repeats. These alleles, known as premutation (PM) alleles, are not associated with intellectual disability, but do confer the risk of a form of ovarian dysfunction known as fragile X-associated primary ovarian insufficiency (FXPOI: OMIM # 311360) [6]. Both male and female PM carriers are also at risk of an adult-onset form of neurodegeneration known as fragile X-associated tremor/ataxia syndrome (FXTAS: OMIM # 300623) [6]. PM pathology is thought to result from the deleterious consequences of having long CGG-repeat tracts in the *FMR1* transcript (reviewed in [7]). The disorders seen in carriers of PM and FM alleles are known collectively as the *FMR1* disorders, the fragile X spectrum disorders, or the fragile X related disorders (FXDs) for the fragile site that once served as a diagnostic feature of FXS [6].

In addition to the expansions that cause these disorders, contractions of the repeat and deletions associated with the loss of flanking sequences are also associated with PM and FM alleles. In some cases, this generates a normal sized allele [8,9,10,11]. In other cases, a deletion involving the repeat and a variable amount of one or both flanking sequences is seen. The consequences of these deletions depend on how far they extend into the 5′ and 3′ flanking regions. Some cases involve a minimal loss of flanking sequence, with a normal phenotype resulting if the repeat number is now in the normal range [12,13,14,15]. Other cases involve a deletion of the entire *FMR1* gene or critical regions of exon 1. This can result in a deficit of FMRP, resulting in a phenocopy of the symptoms seen in FM carriers [8,16]. Contraction events that generate a single allele that is present in all cells presumably occur prezygotically [9,10,15], but contractions can occur post-zygotically, resulting in individuals who are mosaic for different sized alleles [10,11,17,18,19,20]. It has been estimated that 38% of FM carriers are mosaic for additional FM alleles, PM alleles, or both [21]. This mosaicism results in a variable phenotype depending on the proportion of cells that have normal, PM, or FM alleles and, for those individuals with PM alleles, the size distribution of those alleles [22,23,24]. In addition to repeat instability, an increased incidence of the loss of the end of the long arm of the affected X chromosome has been shown in early FXS embryos [25]. A high risk of mosaic Turner syndrome, caused by the loss of the affected X chromosome, has also been reported in female FXS fetuses [26]. Thus, expanded CGG-repeat tracts cause two forms of genetic instability, one involving localized changes in and around the repeat tract, while the second causes chromosome instability, resulting in loss of all or part of the affected chromosome, which is seen in some carriers of FM alleles.

There are several other diseases resulting from unstable CGG-repeat tracts and their associated folate-sensitive fragile sites [27]. The mechanisms of instability and fragility at these loci are likely to be similar to those operating at the *FMR1* locus. These diseases belong to a much larger group of clinical conditions known as the repeat expansion diseases that all result from expansion of a disease-specific short tandem repeat tract [28]. Whether these diseases all share a common expansion mechanism is the subject of much debate, but, as will be discussed later, evidence from common genetic modifiers of expansion risk in some of these diseases and in the mouse models of these diseases suggests that they may do so. Repeat instability and chromosome fragility have been reported in many different organisms and several mechanisms have been proposed to explain these phenomena (see [29,30,31] for excellent recent reviews). It has been suggested that there may be a common underlying mechanism responsible for both expansions and deletions/contractions in the repeat expansion diseases, one that is also shared with chromosome fragility. However, what best explains events occurring at the *FMR1* locus in PM and FM carriers is still an open question. This review will discuss evidence from PM and FM carriers and patients with other repeat expansion diseases that allows us to narrow our focus to a subset of possible mechanisms that account for these events.

## 2. FRAXA Chromosome Fragility

By metaphase, chromosomes are normally fully condensed in preparation for anaphase and cytokinesis. However, at this stage, fragile sites have a microscopic appearance consistent with chromatin that has not yet been condensed. In the case of FRAXA, expression of the fragile site is limited to FM alleles that are transcriptionally silenced [32]. This does not mean that silencing per se is required for fragility. Rather, it may reflect the fact that silenced alleles replicate later in the cell cycle than transcriptionally active ones. Normal alleles replicate late in S phase, while FM alleles replicate in G2/M under normal growth conditions [33]. When replication is further delayed because of folate stress, more cells enter mitosis without having completed replication of the *FMR1* region [33,34].

In contrast to the many fragile sites induced by aphidicolin, a DNA polymerase inhibitor, FRAXA and other fragile sites associated with long CGG-repeat tracts are induced by treatments that negatively impact thymidylate synthase, and thus the size and composition of the nucleotide pools available for replication [35]. CGG-repeats form stable intrastrand structures, including hairpins and quadruplexes, that contain a mixture of base mismatches or non-canonical base interactions [36,37,38,39,40,41,42,43,44]. These structures block DNA synthesis in vitro and are thought to be responsible for the blocks to DNA replication seen in cell models [45] as well as the delayed replication of FM alleles [32,46]. FX cells treated with 5-fluoro-2′-deoxyuridine (FdU), a direct inhibitor of thymidylate synthase, show an increase not only in the expression of the fragile site, but also in the incidence of ultrafine anaphase bridges arising at the *FMR1* locus [47]. These ultrafine bridges stain with DAPI, a DNA stain, but do not contain histones. They are also associated with RPA, a single-stranded DNA binding protein, suggesting the presence of single-stranded DNA. Accumulation of these RPA+ve ultrafine bridges depends on RAD51, a protein essential for homologous recombination. This suggests that the ultrafine bridges represent unresolved homologous recombination intermediates. These ultrafine bridges are seen even in the absence of FdU, indicative of problems at this locus even under normal growth conditions. Examination of binucleated G1 cells shows a high frequency of daughter cells that either lack the FM allele or have a FRAXA-positive micronucleus [47]. Extended growth in FdU results in the high frequency loss of the entire X chromosome carrying the FM allele, linking replication problems, fragility, and aneuploidy [47].

**Figure 1:**
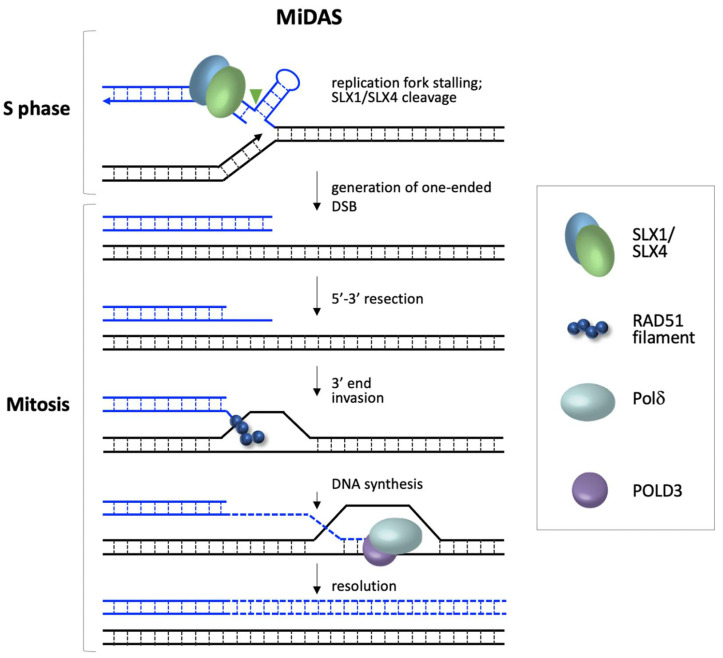
Model for mitotic DNA synthesis (MiDAS) at the *FMR1* locus. FM alleles with stalled replication forks that have not been resolved by the time mitosis begins are cleaved by the SLX1/SLX4 nuclease to generate a one-ended DSB. After end resection to generate a 3′ overhang, binding of RAD51 allows the exposed 3′ end to invade a homologous template, likely a sister chromatid. Conservative DNA synthesis then occurs in a POLD3-dependent fashion to complete replication [48]. Failure to initiate this process results in the formation of ultrafine bridges and the high frequency loss of the affected X chromosome [26,48], while failure to complete MiDAS results in fragile site expression [48].

FM alleles undergo mitotic DNA synthesis (MiDAS) in response to folate stress [48]. MiDAS is thought to be a salvage pathway used by cells to ensure that genomic regions are duplicated before cell division occurs. As illustrated in Figure 1, MiDAS at FM alleles occurs via a RAD52-independent, but RAD51-dependent process that requires POLD3, a subunit of Polδ that is not required for normal DNA replication [48]. The requirement for POLD3 suggests that MiDAS involves a break-induced replication (BIR)-like process. BIR is generally initiated by a one-ended break generated by cleavage of a stalled replication fork, or similar structure, by a structure selective endonuclease. In the case of FM alleles, the endonuclease involved is SLX1/SLX4 [48]. Following cleavage and 5′ to 3′ end resection, RAD51 is thought to bind to the free 3′ single stranded region and facilitate strand invasion, as illustrated in Figure 1 [48]. This is followed by a form of conservative replication that allows this region of the chromosome to be properly duplicated. Inhibition of MiDAS prevents chromosome fragility but increases the frequency of chromosome mis-segregation [48]. It has been suggested that fragility is the result of the delayed chromosome condensation that occurs when MiDAS has been initiated but does not get completed in time [48]. This leaves the chromosome prone to breakage, and that might account for the high frequency of terminal deletions that have been observed in FXS embryos [25]. BIR also involves frequent template-switching. Copy number variations can frequently result because of this process when mispriming occurs within repeats. Chromosome mis-segregation, on the other hand, has been suggested to result from the failure to initiate MiDAS at all [48]. This mis-segregation could account for the high frequency loss of the affected X chromosome in female fetuses carrying a FM [26]. Similar events are likely to be associated with other folate-sensitive fragile sites.

## 3. Repeat Instability

The risk of CGG-repeat expansion at the *FMR1* locus is known to be directly related to the length of the repeat tract and inversely related to the number of AGG interruptions often present at the 5′ end of the repeat tract [21,49,50]. In female PM carriers, expansions outnumber contractions by a factor of 10 to 1 [50] and, when the repeat number approaches 90, there is close to 100% probability of an intergenerational transmission of an allele that now has >200 repeats [50,51]. Increasing maternal age also increases the expansion risk [51]. While FM alleles are only maternally transmitted, at smaller repeat lengths, paternal alleles are prone to expand in gametes [52] and small expansions are more frequent on paternal transmission than on maternal transmission [50].

Once alleles expand into the FM range and become methylated, the repeat tract ceases to be expansion-prone [53,54]. It is also evident that expansion in female PM carriers only occurs when the PM allele is on the active chromosome [55]. These observations suggest that transcription or a euchromatic chromatin configuration is required for repeat expansion. The expansion profiles seen in the blood of females with PM alleles [56] are consistent with mathematical modelling suggesting that expansion proceeds via the frequent gain of a small number of repeats, on average ~1–2 repeats/event [57]. In contrast, large contractions accumulate rapidly in somatic cells in culture [58].

While the hypothesis that contractions and expansions result from the same underlying process is reasonable, there is evidence to suggest that this is not the case. For example, slowing replication with FdU in lymphoblasts from human PM carriers results in contractions, but not expansions [47]. While it is possible that this reflects selection for smaller alleles when cells are subject to replication stress, contractions of silenced FM alleles are readily seen in tissue culture, suggesting that, in contrast to expansions, some contractions do not require transcription [58]. In addition, while most somatic expansions occur via the addition of a small number of repeats with each event, contractions generally result in large and variable changes in repeat number. Furthermore, while AGG interruptions significantly reduce the intergenerational expansion frequency, they have no effect on the frequency of both maternally and paternally transmitted contractions in FX families [50]. Given that fragility is seen only on non-transcribed alleles and expansion is only seen on transcriptionally active alleles, a common trigger for expansion and fragility is also unlikely.

### 3.1. Repeat Expansions

To date, no genome-wide association studies of PM carriers has been done to identify trans-acting genetic factors involved in the CGG-repeat expansion process at the *FMR1* locus. However, expansions in the FXDs share many similarities with other repeat expansion diseases. This includes the small number of repeats gained with each expansion event, their frequency, their dependence on transcription, and the fact that expansion occurs in non-dividing cells. In fact, expansions in non-dividing striatal neurons in Huntington disease can result in the gain of many hundreds of repeats during an individual’s lifetime [59]. Work from some of these diseases, including Huntington disease (HD), myotonic dystrophy type 1 (DM1), and many spinocerebellar ataxias, has implicated some of the proteins involved in mismatch repair (MMR) in the expansion in somatic cells [60,61]. Specifically, MSH3, MLH1, and MLH3, are thought to be involved in promoting expansions, while FAN1, which has recently been shown to also play a role in MMR, has a protective effect [62].

These same proteins play equivalent roles in promoting and protecting against both somatic and germline expansions in a knock-in mouse model of the FXDs [63,64,65,66]. There are a number of other parallels between expansion in this mouse model and instability in PM carriers and individuals with other repeat expansion diseases, including the high frequency of small expansions [55], a requirement for transcription [67], and the fact that expansion occurs in non-dividing cells [68]. It is formally possible that different expansion mechanisms operate in dividing and non-dividing cells. However, in the FXD mouse model, the same genetic factors that are required for expansion in dividing cells are required for expansion in non-dividing ones. Thus, at least in this model, a similar mechanism of expansion likely operates in all expansion-prone cell types.

MSH2, the binding partner of MSH3 in the MutSβ complex, is also required for expansion in the FXD mouse model [69], while MSH6, the MSH2 binding partner in the MutSα complex, plays an important auxiliary role [65]. It has been shown that the nuclease activity of MLH3 is required for expansion [70]. This is interesting because MLH3 is a relatively minor player in MMR, although it plays a critical role in meiosis [71], where it is involved in processing Holliday junctions into crossover products [72]. In addition to MLH3, expansion in the FXD mouse model also requires PMS1 and PMS2 [73]. All three proteins are binding partners of MLH1, forming MutLα (MLH1/PMS2), MutLβ (MLH1/PMS1), and MutLγ (MLH1/MLH3). The requirement for all three MutL complexes is perplexing because they have not been shown to act in concert in MMR. Furthermore, the role of MutLβ is intriguing because, while it is much more abundant than MutLγ, it lacks nuclease activity and has no clearly defined role in MMR. The protective role of both FAN1 and EXO1, another nuclease involved in MMR that also protects against expansion in the mouse model [64], might be explained by competition for the products of MutL cleavage between the canonical MMR pathway, which restores the original allele, and an alternative pathway that leads to expansion.

**Figure 2:**
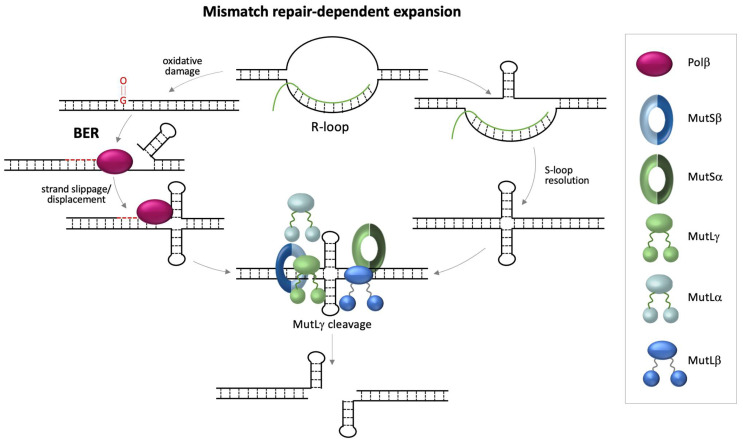
Diagrammatic representation of possible transcription-related, MMR-dependent events that could give rise to repeat expansions. As illustrated on the left-hand side of this figure, R-loops could give rise to expansions because single stranded regions of the R-loop would be prone to oxidative damage. Repair of this damage by BER would create an opportunity for strand slippage and strand-displacement that could result in the formation of hairpins or loop-outs on one or both strands [83]. Alternatively, as illustrated on the right, hairpin formation might occur on the non-template strand of the R-loop, forming an S-loop. This might favor formation of a hairpin on the template strand after dissociation of the transcript. Hairpins or double loop-outs formed by either process could then be bound by both MutS proteins. This results in the recruitment of MutLα, MutLβ, and MutLγ. MutLγ cleavage of the strands opposite each loop-out would generate a DSB that would then be repaired by some, as yet unknown, non-homologous end-joining-independent DSBR pathway.

Why transcription is required for repeat expansion is unknown. One early idea in the field was that it could be related to problems associated with long CGG-repeat tracts that were resolved by transcription coupled repair (TCR), the major DNA repair pathway limited to transcribed regions of the genome [74]. However, CSB, a protein essential for TCR, is not required for expansion in the mouse model [75,76]. More recently, it has been shown that stable R-loops form at the *FMR1* locus [77,78,79,80] and, potentially, these R-loops could be processed into double strand breaks. Because, in the FXD mouse model, non-homologous end-joining, the major double-strand break (DSB) repair pathway operating in mammalian cells, protects against expansion [56], expansion in these animals likely involves a DSB intermediate. However, direct R-loop processing into double-strand breaks is also dependent on CSB [81,82]. R-loops could act as the trigger for expansion in other ways. For example, the single-stranded regions of DNA present in R-loops are likely to be prone to oxidative damage, frequently generating 8-oxo-7,8-dihydroguanine (8-oxoG), the most common product of oxidative damage. As illustrated in Figure 2, such damage may be processed via the base excision repair (BER) pathway, the major pathway for the repair of such damage in mammalian cells. Strand slippage/displacement during long-patch BER may facilitate the formation of hairpins that we have shown to be effectively bound by MutSβ and MutSα in vitro [65]. A role for BER in expansion is suggested by the fact that mutations in OGG1 and NEIL1, DNA glycosylases important for repair of 8-oxoG, reduce expansions in a mouse model of HD [83,84].

As illustrated on the right-hand side of Figure 2, the non-template strand of the R-loop formed at the *FMR1* locus likely forms an intra-strand structure in patient cells [78,80]. Resolution of the R-loop would leave the template strand without a complementary strand with which to hybridize and this could result in the formation of a double loop-out structure that would also be predicted to bind MutSβ and MutSα [65]. Cleavage by MutLγ occurs on the strand opposite the mismatched lesion [85]. Thus, MutLγ processing of double loop-outs formed either during BER or simply by transcription itself could result in the generation of a DSB that is repaired in some way to generate expansions.

A CGG-reporter construct integrated into a murine erythroid leukemia cell line shows both expansions and contractions occurring at approximately equal frequencies. These events depend upon the Polδ subunit POLD3 and the recombination proteins RAD51 and RAD52 [86]. It is probable that these changes arise via some form of BIR that is likely similar to the process shown in Figure 1, except for the fact that RAD52, as well as RAD51, is required. A 3′ single stranded region resulting from a break within the repeat would contain repeats that could potentially anneal out of register on the invaded chromosome or chromatid. This would ultimately generate expansions or contractions depending on whether annealing and subsequent priming occurred upstream or downstream of the breakpoint. Multiple template-switching events, which are known to occur at the early stages of BIR, could also potentially result in the gain or loss of repeats. While BIR is usually involved in the repair of one-ended breaks originating at a stalled replication fork, it has been suggested that BIR can be initiated at double-strand breaks induced in R-loops [87]. Thus, a BIR-dependent process triggered by an R-loop may allow expansion in both dividing and non-dividing cells [88]. However, R-loop mediated BIR has been reported to be CSB-dependent [89]. Furthermore, the BIR associated with FRAXA chromosome fragility is dependent on the SLX1/SLX4 endonuclease to generate the one-ended break [48]. The use of this nuclease to initiate BIR would seem to obviate the need for the MutLγ nuclease. MutLγ does generate double-strand breaks by cleavage of R-loops in yeast [90], so it could be that MutLγ cleavage of R-loops provides the trigger for BIR-mediated repeat expansion. However, this cleavage is independent of the MutS proteins, at least in yeast. BIR-mediated CAG-expansions in yeast are also independent of MSH2, MSH3, and MSH6 [91].

While the loss of MMR proteins such as MLH3 eliminates most, if not all, expansions in mice, it could be argued that, in humans, some expansions arise via an MMR-independent mechanism. In particular, it has been suggested that the large increases in repeat number associated with FM alleles inherited from female PM carriers might arise from a mechanism that generated a large expansion in a single event. BIR has been proposed as just such a mechanism [86]. However, the BIR-dependent expansions in the mouse erythroid leukemia cell reporter system occur at a frequency many orders of magnitude lower than the repeat expansions seen in the FXD mouse model and generally involved the gain of fewer than 30 repeats [86]. Whether the frequency of such events would be high enough in the human oocyte to explain the large gains in repeat numbers seen on maternal transmission of PM alleles is unclear, particularly because contractions, which occurred at a similar frequency and tended to be larger in magnitude than the expansions, would potentially be able to offset some of these gains.

### 3.2. Repeat Contractions

When cells from PM carriers are grown in the presence of FdU, contractions rather than expansions are observed [47]. This might reflect the difficulty of replication through the repeat tract, a phenomenon that would be exacerbated by the reduced availability of nucleotides in FdU-treated cells. As mature sperm in adult men have undergone many more rounds of cell division than oocytes in women of the same age [92], selection for contractions in replicating cells may explain why PM males do not transmit FM alleles and why males with FM alleles in somatic cells have PM alleles in their sperm [93].

**Figure 3:**
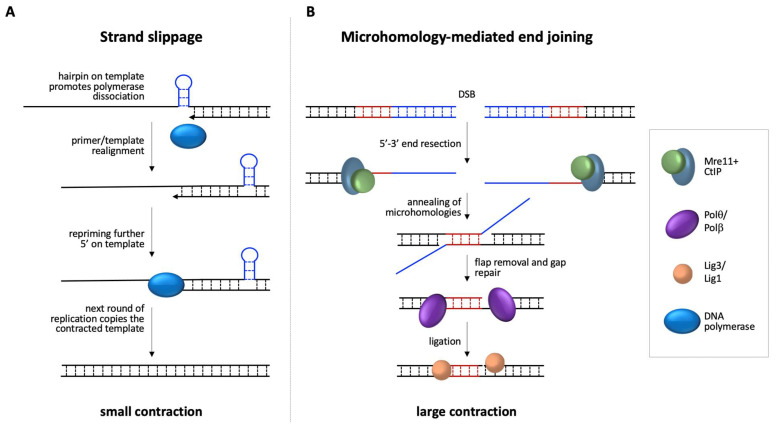
Diagrammatic representation of potential contraction/deletion pathways. (**A**) Strand-slippage during replication may be exacerbated by hairpins formed on the template strand. Repriming more 5′ on the template would lead to nascent strands with fewer repeats than the template strand. A subsequent round of replication would generate a contracted allele. (**B**) MMEJ-mediated repair of a DSB is initiated by end-resection to reveal MHs at either side of the break. Annealing of these MHs is followed by removal of non-homologous flaps, filling of any gaps, perhaps by Polθ or Polβ, and ligation by either ligase 3 (Lig3) or ligase 1 (Lig1).

Silenced FM alleles do contract [20,58], thus some contractions must be transcription independent. Furthermore, the loss of MMR proteins that are required for expansion results in an increase in contractions in the FXD mouse model. These findings reinforce the idea that expansions and at least some contractions arise via different mechanisms. Of interest is the fact that one class of contractions that increases in MMR-deficient cells is associated with the loss of 1–2 repeats. This loss occurs in a large fraction of alleles in mESCs with null mutations in MutLα or MutLγ [73]. These small contractions are likely the products of strand-slippage occurring during replication that are normally repaired by MMR, as illustrated in Figure 3A. This might suggest that most strand-slippage events are small and that larger contractions occur via a different mechanism.

Notably, most of the breakpoints of larger deletions seen in the children of PM and FM carriers that have been characterized at the sequence level are associated with microhomologies (MHs) of 2–9 nucleotides [8,12,13,14,15,16,23,94,95,96,97,98,99,100,101]. Despite the GC-richness of the *FMR1* locus that reduces the sequence space, it is possible that such MHs reflect the mechanism involved in the generation of these deletions. Such MHs are the hallmarks of a process known as microhomology mediated end joining (MMEJ). As illustrated in Figure 3B, the process of MMEJ involves end-resection of the DSB by Mre11 and CtIP, followed by a search for short MHs in the two DNA single-stranded tails, annealing of the regions of microhomology, removal of the non-homologous flaps, gap filling, and finally ligation.

Breaks with minimal resection could result in deletions within the repeat (contractions), while more extensive resection would result in deletions that extend into the flanking regions. MMEJ is often accompanied by nucleotide insertions at break sites. The insertions are often derived from sequences close to the breaks or are added de novo because of non-templated extension of the 3′-termini of breaks. These insertions are characteristic of Polθ, the major polymerase implicated in MMEJ [102]. A 7 bp insertion associated with a deletion that extended both upstream and downstream of the repeat has been reported in the son of a mother with a FM allele [8]. In addition, three examples of the de novo generation of an *EagI* site associated with a contraction of the repeat have been reported in humans [103,104,105]. As the *EagI* recognition site is CGGCCG, the new site could arise either from a base substitution or from the insertion of a CCG triplet. We have observed the de novo generation of a *SmaI* site (CCCGGG) within the repeat in our FXD mouse model that might have arisen the same way (Zhao and Usdin, unpublished observations). Thus, MMEJ and Polθ-mediated end-joining, in particular, may be an appealing candidate for the process that generates contractions or deletions in PM and FM carriers.

## 4. Concluding Remarks

**Figure 4:**
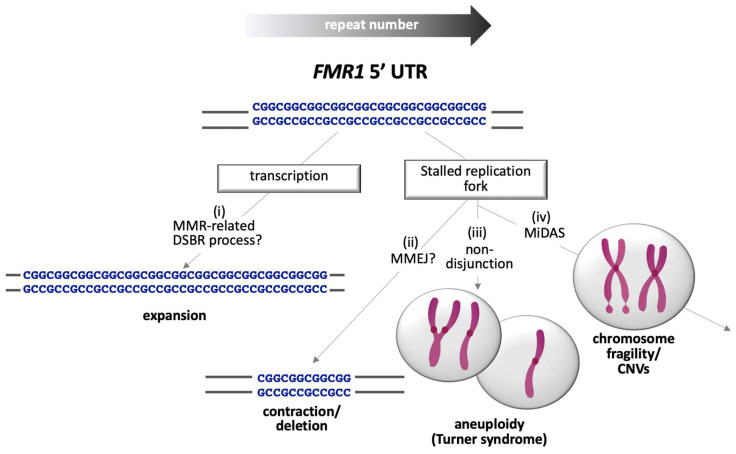
Summary of potential sources of genomic instability associated with PM and FM alleles. Transcription of long CGG-repeat tracts promotes a form of MMR-dependent DSBR that leads to expansion (i). As the repeat number increases, the incidence of replication fork stalling increases. Repair of the stalled fork can lead to contractions or deletions (ii). Aneuploidy can result when stalled forks are not repaired (iii), while chromosome fragility results when repair of the stalled forks by MiDAS is incomplete (iv).

In summary, expanded repeats at the *FMR1* locus represent a triple threat to the genome. As illustrated in Figure 4, one threat is present in the form of repeat expansion that leads to the FXDs, the second in the form of deletions that can phenocopy the symptoms of FXS, and the third in the form of chromosomal structural and numerical abnormalities that can result from problems associated with replication of the repeats including fragile site expression.

These threats are not limited to the *FMR1* locus, but, by extension, are likely to be relevant to the many other CGG-repeat tracts that are seen in human genomes. While the current data indicate that the sources of genetic instability at the *FMR1* locus, expansion, contractions/deletions, and chromosome fragility result from different problems caused by the expanded CGG repeat tract, more work is needed to better understand the mechanisms responsible and what, if any, overlap there is in the pathways involved. While, at this point, there is no evidence for a role of MMR in chromosome fragility, the bulk of the evidence points to an important role for MMR proteins in many, if not all, expansions, both in the FXDs and in other repeat expansion diseases. This is relevant as there are some encouraging signs that it may be possible to reduce MMR-dependent expansions by targeting components of the MMR machinery like PMS1 that are not associated with significantly increased cancer risk [106].

## Data Availability

Not applicable.
